# How generative AI becomes embedded in design learning: a 14-instructional-day intensive longitudinal study of reciprocal dynamics between AI use and self-efficacy

**DOI:** 10.3389/fpsyg.2026.1975841

**Published:** 2026-10-14

**Authors:** Yang Li, Yi Yang, Silu Bao

**Affiliations:** 1 School of Media, Guangxi Polytechnic University, Nanning, China; 2 School of Design, Jiangnan University, Wuxi, China; 3 Faculty of Information Engineering, Xinjiang Institute of Technology, Aksu, China

**Keywords:** design learning, diary study, dynamic structural equation modeling, generative AI, self-efficacy

## Abstract

Generative AI is increasingly embedded in design education, yet little is known about how students’ daily engagement with generative AI is dynamically associated with their learning-related capability beliefs across successive instructional days. Drawing on self-efficacy theory, this study examined the reciprocal next-instructional-day associations between daily generative AI use in design learning and daily design-learning self-efficacy, and further investigated whether baseline hassles were associated with individual differences in these dynamics. A 14-instructional-day diary study was conducted with 260 Chinese undergraduate design students, yielding 3,558 observed person-day assessments out of 3,640 possible assessments. Daily generative AI use and design-learning self-efficacy were assessed throughout the diary period, whereas hassles were measured at baseline. The data were analyzed using bivariate dynamic structural equation modeling within a Bayesian estimation framework. The results showed significant autoregressive effects for daily generative AI use (*β* = 0.198, 95% CrI [0.154, 0.237]) and daily design-learning self-efficacy (*β* = 0.138, 95% CrI [0.100, 0.175]). At the within-person level, higher-than-usual generative AI use on one instructional day positively predicted design-learning self-efficacy on the subsequent instructional day (*β* = 0.043, 95% CrI [0.006, 0.077]), while higher-than-usual design-learning self-efficacy also positively predicted generative AI use on the subsequent instructional day (*β* = 0.059, 95% CrI [0.023, 0.095]). At the between-person level, greater baseline hassles were associated with higher average generative AI use (*β* = 0.135), lower average design-learning self-efficacy (*β* = −0.254), and a stronger cross-lagged association from generative AI use to subsequent self-efficacy (*β*= 0.458). Gender did not significantly predict the main random effects. These findings indicate modest reciprocal next-instructional-day associations between generative AI use and design-learning self-efficacy, suggesting that the two may reinforce one another across instructional days. The stronger association from generative AI use to subsequent self-efficacy among students reporting greater baseline hassles further suggests that contextual demands may shape how generative AI becomes integrated into students’ day-to-day design learning.

## 1 Introduction

The rapid adoption of generative AI in higher education has shifted scholarly attention from whether students use AI tools to how such tools are incorporated into everyday learning practices. Tools such as ChatGPT, Gemini, Claude, Midjourney, and Stable Diffusion can provide students with instant feedback, conceptual explanations, text generation, visual references, design suggestions, and personalized learning support, and are therefore regarded as having the potential to support learning, improve learning efficiency, and promote learning engagement (Chan and Hu, 2023; Crompton and Burke, 2023; Kasneci et al., 2023). Recent studies have further shown that university students widely use generative AI for activities such as content summarization, clarification of complex concepts, writing assistance, task planning, and learning feedback, and generally perceive its value in time management and learning support (Cena et al., 2026; Farinosi and Melchior, 2025). At the same time, the use of generative AI in education has raised concerns regarding academic integrity, overreliance, weakened critical thinking, information accuracy, ethical boundaries, and the transfer of learning responsibility (Cotton et al., 2024; Farrokhnia et al., 2024; Lo, 2023; Tlili et al., 2023). Therefore, a key question in current research is not only whether students use generative AI, but how such use becomes connected to their learning-related beliefs in specific disciplinary contexts.

Recent evidence from adjacent AI and human–computer interaction contexts further indicates that AI-mediated support does not produce uniformly positive outcomes but may shape users’ judgments and psychological responses in context-dependent ways. For example, AI-generated review summaries can reduce decision satisfaction when generated information systematically biases users’ expectations (Wang Y. et al., 2026), whereas AI-guided support has been shown to improve emotional clarity, coping, and stress-related outcomes compared with unguided journaling (Wang R. et al., 2026). Research in technology-mediated virtual environments likewise suggests that emotional engagement and subsequent behavior are sensitive to specific interface and self-representation cues (Huang et al., 2025). Together, these findings underscore the importance of examining how particular forms of AI and digital technology use become linked to subsequent psychological states rather than assuming a uniform technological effect.

This issue is particularly important for university students majoring in design. Design students provide a theoretically meaningful context for examining the subsequent instructional day role of generative AI because design learning is rarely limited to the acquisition of fixed knowledge. Instead, students are expected to frame ambiguous problems, search for visual and conceptual references, generate alternative solutions, receive feedback, revise their work, and gradually transform abstract ideas into visual or material outcomes. In this context, generative AI may function not only as an information tool, but also as a design-learning scaffold that helps students move from uncertainty to temporary task progress. This makes design students an especially relevant group for examining whether AI-supported task progress is associated with changes in students’ learning self-efficacy across subsequent instructional day.

Design learning is typically open-ended, process-oriented, and iterative, requiring students to move continuously through problem understanding, inspiration seeking, concept generation, alternative comparison, visual expression, feedback-based revision, and work refinement. Design research has long emphasized problem framing, reflective practice, design thinking, inspiration acquisition, and iterative development in design activities (Cross, 2001; Dorst, 2011; Vasconcelos and Crilly, 2016). Compared with standardized knowledge learning, design learning depends more heavily on students’ ability to sustain progress in uncertain and ill-defined tasks. Generative AI is especially suited to this learning context because it can support idea generation, visual referencing, alternative exploration, textual explanation, and expression refinement. However, existing higher education research on generative AI has mainly focused on general university students or general learning tasks, leaving limited evidence on how generative AI is used in creative, project-based, and iterative design learning contexts.

When examining the learning consequences of generative AI use, design learning self-efficacy represents an important psychological variable. Self-efficacy refers to individuals’ beliefs about their capability to organize and execute specific actions required to attain desired goals (Bandura, 1977, 1997). A substantial body of research in learning contexts has shown that self-efficacy is closely associated with academic motivation, learning persistence, self-regulation, and academic performance (Honicke and Broadbent, 2016; Schunk and DiBenedetto, 2020). Recent evidence from Chinese higher education further underscores the relevance of efficacy beliefs. Tang et al. (2026) showed that academic self-efficacy formed part of multiple configurations associated with academic achievement among Chinese undergraduates, indicating that efficacy beliefs operate together with contextual support in shaping academic outcomes. This finding provides additional support for examining self-efficacy as an important learning-related belief in the Chinese undergraduate context. In the context of design learning, design learning self-efficacy reflects students’ confidence in completing design tasks, generating design ideas, solving design problems, revising their work based on feedback, and advancing course projects. Unlike general academic self-efficacy, design learning self-efficacy involves students’ confidence in handling ill-defined problems, tolerating ambiguity, generating original ideas, incorporating feedback, and transforming abstract concepts into visual or material outcomes. Because design tasks are often characterized by high uncertainty, open-ended assessment criteria, and repeated revision requirements, students’ self-efficacy may fluctuate with their daily learning experiences. Recent research on digital technology-supported learning has also shown that students’ technology attitudes, digital literacy, and technological self-efficacy influence their learning engagement, while AI-supported learning may shape students’ learning experiences through feedback, scaffolding, and cognitive load regulation (Getenet et al., 2024; Hartelt and Martens, 2024; Jia and Tu, 2024).

Generative AI use and design learning self-efficacy may have a bidirectional relationship. On the one hand, generative AI use may enhance subsequent design learning self-efficacy. According to self-efficacy theory, individuals’ beliefs about their capabilities are shaped by sources of information such as mastery experiences, vicarious experiences, social persuasion, and emotional states (Bandura, 1997; Usher and Pajares, 2008). In design learning, generative AI can help students quickly obtain inspiration, generate alternative solutions, compare visual expressions, refine design descriptions, and receive immediate feedback. Such support may reduce uncertainty in the early stages of ideation, making it easier for students to develop a sense of task control and confidence in their abilities. On the other hand, design learning self-efficacy may also promote subsequent generative AI use. Students with higher self-efficacy are generally more willing to engage in complex tasks, try new strategies, and persist in problem solving. Thus, when students feel more confident on a given instructional day about their ability to complete design tasks, they may be more likely the next day to actively explore AI tools, refine prompts, compare generated outputs, and integrate AI-generated content into their own design thinking. In other words, generative AI use and design learning self-efficacy may not be linked through a unidirectional effect, but may instead show a reciprocal pattern across consecutive learning days.

The motivation for the present study arises from three closely related considerations. First, generative AI is increasingly becoming part of students’ everyday design-learning activities, yet it remains unclear “whether engagement with these tools across successive instructional days is associated with subsequent changes in students’ confidence in managing uncertain and iterative design tasks. This question is particularly relevant in design education because students must repeatedly generate ideas, compare alternatives, respond to feedback, and revise their work, making their perceived capability likely to fluctuate with daily task experiences (Cross, 2001; Dorst, 2011; Vasconcelos and Crilly, 2016). Second, the relationship may be reciprocal rather than unidirectional. Generative AI may provide task support that contributes to subsequent efficacy beliefs, while students with stronger efficacy beliefs may in turn be more willing to actively explore and integrate AI tools into their learning activities (Bandura, 1997; Chan and Hu, 2023; Getenet et al., 2024). Third, these dynamics may differ across students depending on their broader task-demand context. Students experiencing greater baseline hassles may rely more strongly on external support when progressing through demanding design tasks, while at the same time reporting lower learning confidence (Tachon et al., 2021; Liu et al., 2024). Examining these processes at the daily within-person level is therefore important for understanding not merely whether AI use and self-efficacy are associated across students, but how the two may become dynamically linked within the same student over time.

Although research on AI and higher education has grown rapidly, several limitations remain. First, existing studies have largely examined the relationships between AI use and learning outcomes, learning attitudes, or technology acceptance from a cross-sectional perspective, with limited attention to how students’ AI use and psychological states co-vary over time in daily learning processes. Second, prior research has typically focused on general university students or general learning tasks, giving insufficient attention to design students as a distinctive learning group. Third, AI use has often been treated as either a unidirectional predictor or outcome variable, while little research has examined whether AI use and learning self-efficacy may form a subsequent instructional day, bidirectionally reinforcing relationship. Recent studies have begun to emphasize students’ real-world experiences with generative AI and its ethical boundaries (Cena et al., 2026; Farinosi and Melchior, 2025), while related research has also highlighted the roles of digital literacy, technology-use patterns, AI-mediated decision making, and psychological responses to AI-supported interaction (Jiang et al., 2023; Wang Y. et al., 2026; Wang R. et al., 2026). Intensive longitudinal research further suggests that short-term psychological states and more stable individual characteristics may be differently associated with daily technology-use behavior (Chu et al., 2025). Nevertheless, empirical evidence remains limited regarding whether generative AI use and learning-related self-efficacy dynamically predict one another within the same student across subsequent instructional day, particularly in project-based and iterative design-learning contexts. These gaps indicate the need for an intensive longitudinal approach that can distinguish within-person fluctuations from stable between-person differences and examine reciprocal temporal associations in a discipline-specific learning context.

Therefore, this study focuses on Chinese university students majoring in design and employs a 14-instructional-day diary design and bivariate dynamic structural equation modeling (DSEM) to examine the dynamic relationship between daily generative AI use in design learning and daily design-learning self-efficacy. Intensive longitudinal data make it possible to capture within-person processes that unfold over time, while DSEM can simultaneously distinguish within-person dynamic relationships from stable between-person differences (Asparouhov et al., 2018). Specifically, the present study has three primary objectives: (1) to examine whether higher-than-usual generative AI use in design learning on a given instructional day predicts higher design-learning self-efficacy on the subsequent instructional day; (2) to examine whether higher-than-usual design-learning self-efficacy on a given instructional day predicts greater generative AI use in design learning on the subsequent instructional day; and (3) to examine whether individual differences in baseline hassles are associated with variation in these daily dynamic relationships, while controlling for gender. By separating within-person fluctuations from between-person differences, this approach enables the present study to determine whether generative AI use and design-learning self-efficacy are dynamically linked within the same student rather than merely correlated across different students.

This study makes three contributions. First, it extends research on generative AI in education from between-person associations to within-person daily processes by examining how AI use and design learning self-efficacy predict each other across subsequent instructional day. Second, it situates generative AI use in design education, a project-based and iterative learning context in which students use AI for ideation, visual referencing, solution comparison, and work refinement. Third, it applies bivariate DSEM to distinguish stable between-person differences from within-person fluctuations, thereby providing more precise evidence on the subsequent instructional day dynamics of generative AI-supported learning.

## 2 Literature review and hypothesis development

### 2.1 The predictive effect of generative AI use on design learning self-efficacy

Generative AI use in design learning refers to students’ daily use of generative AI tools to support ideation, information referencing, solution generation, prompt refinement, work optimization, and feedback acquisition during the design learning process. According to self-efficacy theory, individuals’ efficacy beliefs are primarily shaped by mastery experiences, vicarious experiences, social persuasion, and emotional states (Bandura, 1977, 1997; Usher and Pajares, 2008). However, existing research on generative AI and design learning has mainly focused on students’ perceptions and acceptance of AI-assisted design courses (Jiang et al., 2024); intentions to use AIGC tools (Vail and Chen, 2026); AI-supported ideation and creativity, creative cognition, and learning or design task performance (Hwang and Wu, 2025). Less attention has been paid to the predictive effect of generative AI use on subsequent design learning self-efficacy from the perspective of daily learning processes.

Existing studies provide preliminary support for this inference. Chan and Hu (2023) found that students generally perceive generative AI as capable of providing personalized learning support, assisting brainstorming, and supporting research and analysis. Kasneci et al. (2023) also noted that large language models may enhance learning experiences by generating learning content, facilitating interaction, and supporting personalized learning. Jeon and Lee (2023) further conceptualized ChatGPT as a conversational partner, content provider, teaching assistant, and evaluator in learning, highlighting its role in feedback and learning support. These studies suggest that generative AI may help students advance learning tasks more efficiently by providing immediate feedback, alternative solutions, and interactive support.

When students use generative AI more frequently for design learning on a given instructional day, they may gain more task-related progress experiences, design references, alternative examples, and positive feedback. These experiences can reduce the uncertainty associated with open-ended design tasks and strengthen students’ sense of control over ideation, revision, and work completion. Based on the above theoretical and empirical evidence, this study proposes that higher-than-usual generative AI use in design learning on a given instructional day will positively predict design-learning self-efficacy on the subsequent instructional day.

### 2.2 The predictive effect of design learning self-efficacy on generative AI use

Design learning self-efficacy refers to students’ beliefs in their ability to understand, complete, and improve design learning tasks. According to social cognitive theory, self-efficacy influences not only individuals’ confidence in a task but also their task choices, effort investment, persistence, and strategy use (Bandura, 1997; Schunk and DiBenedetto, 2020; Zimmerman, 2000). Therefore, higher design learning self-efficacy may not only be an outcome of generative AI use, but may also serve as an important predictor of students’ subsequent active use of generative AI.

Existing research provides indirect support for this inference. Studies have shown that students’ use and acceptance of AI tools are not determined solely by technological availability, but are jointly influenced by individual psychological characteristics, AI literacy, learning skills, perceived learning benefits, and specific learning contexts. More broadly, research among Chinese youth and adolescents has shown that digital-technology outcomes are related not only to whether individuals use technology, but also to their Internet literacy, purposes of use, and duration of use (Jiang et al., 2023). This distinction is relevant to generative AI-supported learning because students may differ not only in their access to AI tools but also in how and why they incorporate these tools into specific learning activities. For example, self-efficacy and curiosity have been found to be associated with students’ AI use (Schutte and Li, 2025), while AI literacy and 21st-century skills influence university students’ acceptance of generative AI (Salhab and Aboushi, 2025). Students’ use of ChatGPT also varies across learning tasks, usage purposes, and group characteristics (Ravšelj et al., 2025). In the context of design education, acceptance of AIGC-assisted courses is further shaped by factors such as technological application, instructional content, teaching methods, and extracurricular learning support (Jiang et al., 2024).

In the context of generative AI-supported design learning, the effective use of AI tools involves more than simply entering a question and accepting an answer. Students need to clarify design goals, formulate effective prompts, evaluate the quality of AI outputs, compare alternative solutions, and transform AI-generated content into design outcomes that meet course requirements and reflect their personal expression. Students with higher self-efficacy are generally more willing to actively explore task resources, try new learning strategies, and maintain engagement when facing uncertain outcomes. Therefore, when students report higher design learning self-efficacy on a given instructional day, they may be more likely to use generative AI more actively and frequently to support design learning on the subsequent instructional day. Based on the above theoretical and empirical evidence, this study proposes that higher-than-usual design-learning self-efficacy on a given instructional day will positively predict generative AI use in design learning on the subsequent instructional day.

### 2.3 Between- and within-person associations

Existing research on the educational applications of generative AI has mainly examined between-person associations, that is, whether students who use generative AI more frequently report higher learning motivation, technology acceptance, creativity, or learning performance. Although these studies provide important evidence for understanding the educational value of generative AI, their ability to explain daily design learning processes remains limited. This is because between-person associations cannot reveal how the same student changes across different time points. In other words, between-person associations address whether students who use generative AI more frequently generally have higher design learning self-efficacy, whereas within-person associations address whether the same student shows higher design learning self-efficacy on the subsequent instructional day after using generative AI more than usual on a given instructional day. Methodological research has noted that between-person differences and within-person changes differ in both theoretical meaning and statistical interpretation, and that conclusions based on between-person differences cannot be simply generalized to within-person dynamic processes (Curran and Bauer, 2011; Molenaar, 2004). Recent intensive longitudinal research further demonstrates the importance of distinguishing short-term fluctuations from more stable individual differences when examining technology use. Using a one-month daily diary design combined with objective smartphone-use tracking, Chu et al. (2025) found no direct association between daily negative affect and smartphone use, whereas longer-term psychological distress was associated with different patterns of smartphone use depending on individuals’ locus of control. These findings illustrate that technology-use behavior may be linked differently to short-term psychological states and more stable individual characteristics. However, comparable evidence remains limited regarding reciprocal associations across successive instructional days between generative AI use and learning-related efficacy beliefs, particularly in design education.

In the present study, both generative AI use in design learning and design learning self-efficacy may fluctuate from day to day. Whether students use generative AI more frequently on a given instructional day may be influenced by factors such as design task difficulty, assignment deadlines, feedback needs, inspiration needs, and daily learning pressure. At the same time, students’ design learning self-efficacy may also change with daily task progress, mastery experiences, failure experiences, or feelings of uncertainty. Therefore, revealing the within-person association between these two variables at the daily level can help provide a more accurate understanding of whether generative AI use and design learning self-efficacy show a reciprocal subsequent instructional day association.

Dynamic structural equation modeling provides an appropriate method for addressing this issue. DSEM integrates multilevel modeling, time-series modeling, and structural equation modeling, enabling the simultaneous estimation of within-person autoregressive effects, cross-lagged effects, and between-person random-effect differences (Asparouhov et al., 2018; McNeish and Hamaker, 2020). This approach allows generative AI use and design learning self-efficacy to be analyzed simultaneously as both predictors and outcomes, thereby testing their bidirectional dynamic relationship in daily time series. Wang et al. (2024) used DSEM to examine the daily bidirectional relationship between state coping self-efficacy and state self-compassion, and their findings indicate that DSEM is suitable for testing whether two daily state variables form a mutually reinforcing process. Following this methodological logic, the present study extends DSEM to the context of generative AI-supported design learning and further examines whether daily generative AI use in design learning and daily design learning self-efficacy form a bidirectional dynamic relationship.

In addition, drawing on prior diary studies and research on generative AI in education, this study includes gender and hassles as covariates in the model. Gender is a common individual background variable in educational technology research, and students of different genders may differ in their frequency of generative AI use, confidence in using AI tools, perceived benefits, risk concerns, and technology acceptance (Møgelvang et al., 2024; Ravšelj et al., 2025). Gender was included as a demographic control on theoretical grounds rather than on the basis of its statistical significance in the present sample. Although age and study year were recorded as demographic characteristics, they were not specified *a priori* as covariates or moderators of the focal within-person dynamics. In addition, specific design specialization was not systematically assessed in a manner that would permit reliable incorporation into the present DSEM. Therefore, these variables were not added *post hoc* to the model.

Hassles refer to minor stressors that students repeatedly experience in daily learning and life, such as course-related pressure, time conflicts, interpersonal difficulties, difficulties in progressing with assignments, or frustration arising from design tasks. Previous research has indicated that hassles can influence university students’ stress experiences, psychological states, and life satisfaction (Tachon et al., 2021), while stress levels are also closely related to self-efficacy (Liu et al., 2024).

In the present study, baseline hassles were conceptualized as a between-person indicator of students’ relatively stable task-demand context rather than as a daily time-varying stressor. From the perspective of self-efficacy theory, students experiencing greater hassles may have fewer psychological and task resources available for maintaining progress in uncertain design tasks and may therefore report lower learning confidence. Under such conditions, the immediate task support provided by generative AI, such as idea generation, reference acquisition, solution comparison, and feedback, may become particularly salient. AI-supported task progress may therefore be more strongly translated into subsequent self-efficacy among students experiencing higher levels of baseline hassles. Therefore, gender was treated as a demographic control, whereas hassles were used to capture students’ task-demand context and to examine whether this context shaped average levels and dynamic associations between generative AI use and design learning self-efficacy.

### 2.4 The present study

Overall, this study aims to examine the longitudinal dynamic relationship between daily generative AI use in design learning and daily design-learning self-efficacy among Chinese university students majoring in design. Specifically, it adopts a 14-instructional-day diary design and applies a bivariate dynamic structural equation model (DSEM) to analyze the autoregressive and cross-lagged effects between these two state variables across successive instructional days. Rather than simply asking whether students who use generative AI more frequently report higher design learning self-efficacy, this study further examines whether, within the same student, higher-than-usual generative AI use in design learning on a given instructional day predicts higher design learning self-efficacy on the subsequent instructional day, and whether higher-than-usual design learning self-efficacy on a given instructional day predicts greater generative AI use in design learning on the subsequent instructional day.

Based on the literature reviewed above, the present study addresses three primary objectives. First, it examines whether higher-than-usual daily generative AI use in design learning predicts higher design-learning self-efficacy on the subsequent instructional day. Second, it examines whether higher-than-usual daily design-learning self-efficacy predicts greater generative AI use in design learning on the subsequent instructional day. Third, it examines whether baseline hassles are associated with individual differences in these dynamic relationships, while gender is included as a demographic control variable. In addition to these primary objectives, the model estimates the autoregressive effects of both daily variables to account for their stability across successive instructional days or inertia. Together, these analyses allow the study to distinguish reciprocal within-person dynamics from stable between-person differences. Based on self-efficacy theory and prior research on the educational applications of generative AI, this study hypothesizes that daily generative AI use in design learning and daily design-learning self-efficacy have a positive bidirectional predictive relationship. Because the autoregressive effects and the effects of gender and baseline hassles were exploratory, no directional hypotheses were proposed for these effects.

## 3 Method

### 3.1 Participants

Participants were undergraduate students majoring in design-related disciplines at Chinese universities. A total of 1,100 students completed the baseline survey, and 312 agreed to participate in the subsequent 14-instructional-day diary phase. Participants were included in the analysis only if they met the following criteria: (a) they were undergraduate students in design-related majors; (b) they owned a personal smartphone and were able to complete the online diary survey continuously; (c) they had provided informed consent; and (d) they completed the required proportion of daily follow-up questionnaires.

Among the 312 students who agreed to participate in the daily follow-up survey, we further excluded invalid respondents (*n* = 22), participants whose longitudinal study IDs could not be matched with the baseline survey (*n* = 5), and those with missing covariate data (*n* = 25). Ultimately, data from 260 participants were included in the formal analysis. The detailed sample screening process is presented in Figure 1. For detecting a small within-person effect (γ10.std. = 0.10, ICC = 0.50, *α* = 0.05), at least 131 individuals and 1,834 data points are required (Wang et al., 2024). The present study included 260 participants, who could contribute a maximum of 3,640 person-day assessments across the 14-instructional-day diary period. Of these, 3,558 person-day assessments were completed, exceeding the minimum number of data points required for the planned DSEM analysis. Thus, the sample size was sufficient for the planned analysis.

**Figure 1:**
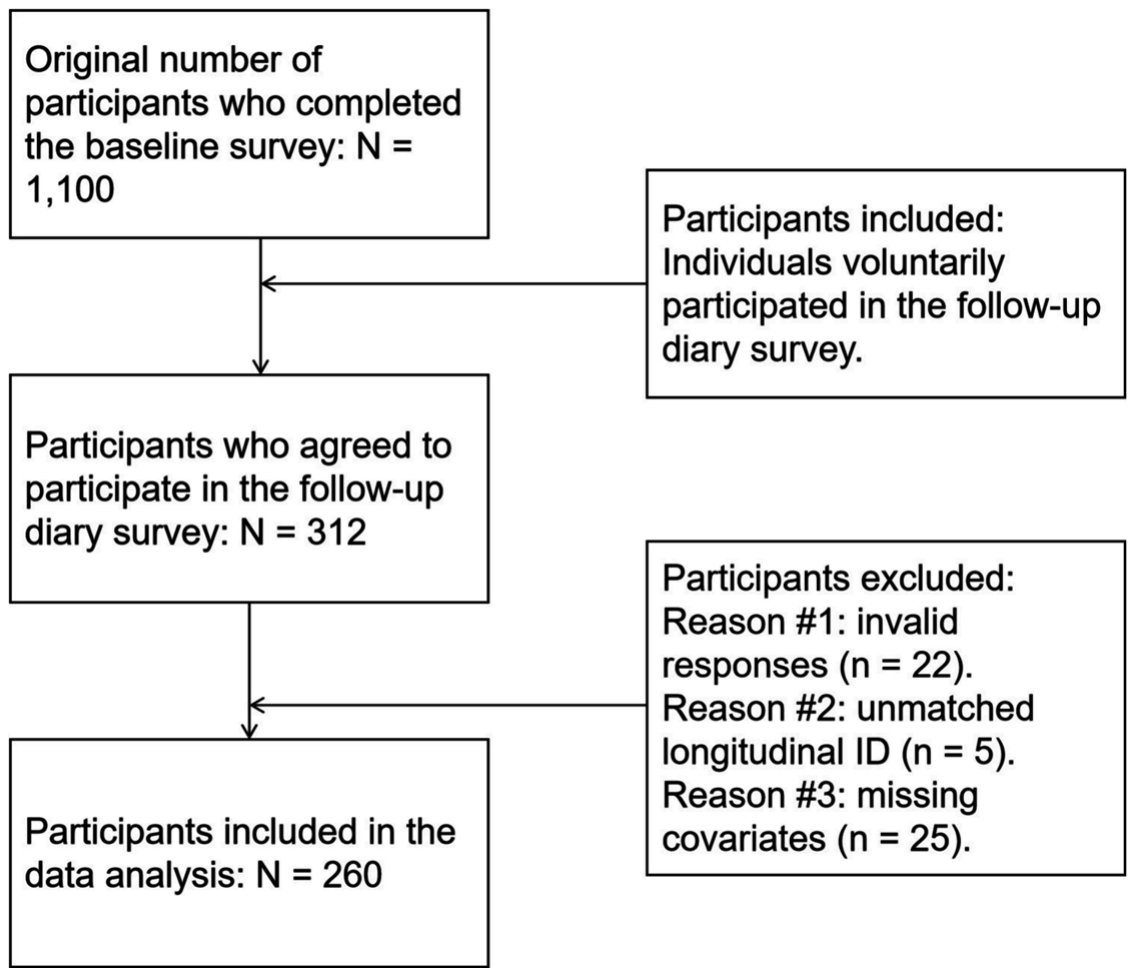
Participant screening process and final analytic sample.

The final sample consisted of 136 male students (52.3%) and 124 female students (47.7%). The participants had a mean age of 20.45 years (SD = 1.04, range = 18–23 years). In terms of grade distribution, 64 participants were Freshman students (24.6%), 67 were sophomore students (25.8%), 65 were junior students (25.0%), and 64 were senior students (24.6%). The demographic characteristics of the final analytic sample are summarized in Table 1.

**Table 1:** Demographic characteristics of the final analytic sample.

Sample	Category	Number	Percentage
Gender	Male	136	52.3%
Grade	Female	124	47.7%
	Freshman	64	24.6%
	sophomore	67	25.8%
	junior	65	25.0%
	senior	64	24.6%
Age (years)	Mean ± SD	20.45 ± 1.04	
	Range	18–23	

### 3.2 Procedures

After eligibility screening and informed consent, participants completed a baseline questionnaire via Wenjuanxing. The baseline questionnaire was used mainly to collect demographic information, baseline hassles, and other study-related background information. Three days after the baseline survey, students who agreed to continue participating in the daily follow-up study were invited to join a research WeChat group to receive daily questionnaire reminders and survey links.

To protect participants’ privacy and match data across different study phases, the research team assigned each participant a fixed study ID. Participants used this ID for identification when completing the daily questionnaires, rather than providing their personal names. The daily follow-up survey was conducted over 14 successive instructional days. Weekends were excluded from the survey schedule, and data collection resumed on the next instructional day after each weekend. On each scheduled instructional day, the research team sent the questionnaire link via the WeChat group at 8:30 p.m. Participants were encouraged to complete the survey promptly after receiving the link, and the questionnaire typically required approximately 10 min to complete. The 10 core daily items were presented in a randomized order across the repeated instructional-day assessments to reduce systematic item-order effects. The relatively high diary-completion rate may have been facilitated by the structured follow-up procedure. Participants received the survey link through the same WeChat group at a fixed time on each scheduled instructional day, providing a consistent reminder. In addition, participants who completed all scheduled surveys received RMB 60 as compensation. Participation remained voluntary, and participants were informed that they could withdraw from the study at any time.

Participants reported their generative AI use in design learning and design-learning self-efficacy for that instructional day. Baseline hassles had been assessed once before the diary phase and were not repeatedly measured during the follow-up period.

Across the 260 participants included in the final analytic sample, 3,558 of the 3,640 possible instructional-day assessments were completed, corresponding to an overall diary-completion rate of 97.75%. A total of 82 assessments (2.25%) were missing across 30 participants. Overall, 230 participants completed all 14 scheduled assessments, and participants completed an average of 13.68 of the 14 scheduled assessments. Additional diary data-quality checks, including questionnaire completion duration, submission-time distribution, and repetitive response patterns across successive assessments, are reported in Supplementary Table S1 in Appendix A.

The 14 instructional-day period was selected to capture repeated fluctuations in design-learning activities across successive instructional days while avoiding systematic differences in formal learning opportunities associated with weekends. For clarity, “daily” refers to each scheduled instructional-day diary assessment rather than to every calendar day.

### 3.3 Measures

#### 3.3.1 Baseline measures

##### 3.3.1.1 Hassles

This study used the Inventory of College Students’ Recent Life Experiences (ICSRLE; Kohn et al., 1990) to assess participants’ baseline level of life hassles during the month preceding the diary study. The scale includes 49 items covering common sources of stress in university students’ academic and daily lives, including academic challenges, time pressure, social alienation, daily irritations, negative social experiences, friendship problems, and romantic relationship problems. Given that design students often face multiple task demands related to course projects, work revision, time management, and interpersonal collaboration, baseline hassles were used to represent participants’ relatively stable stress and task-demand context before entering the diary phase. Participants rated each item based on their experiences during the preceding month on a 7-point scale ranging from 1 (“not at all part of my life”) to 7 (“very much part of my life”). Item scores were averaged, with higher scores indicating greater recent life hassles. The scale showed good internal consistency in the present study (Cronbach’s *α* = 0.92).

#### 3.3.2 Daily measures

##### 3.3.2.1 Daily generative AI use in design learning

Daily generative AI use was operationalized as students’ self-reported daily engagement with generative AI for design-learning purposes. This study used five items developed with reference to research on generative AI-supported learning in higher education, students’ perceptions of AI use in learning, and design knowledge management, and adapted them to the daily learning task context of design students (Chan and Hu, 2023; Chan and Zhou, 2023). The items assessed whether and to what extent generative AI was incorporated into design-learning activities on a given instructional day, including supporting design-learning tasks, obtaining design inspiration or visual references, generating or comparing design solutions, revising and optimizing design work, and supporting design ideation and expression. A sample item was: “Today, I used generative AI to support my design learning tasks.” All items were rated on a 7-point Likert scale ranging from 1 (“strongly disagree”) to 7 (“strongly agree”), and the mean score of the five items was used as the daily generative AI use score. Higher scores reflected greater daily task-related engagement with generative AI in design learning. Importantly, the measure captured the extent of task-related AI engagement rather than the quality or criticality of that engagement. It did not distinguish reflective and evaluative use, such as critically comparing, revising, or transforming AI-generated outputs, from more superficial forms of use, such as directly accepting or reproducing AI-generated content without further evaluation. The measure did not assess objective usage duration, the number of usage episodes, or tool-specific usage logs.

This study first calculated the pooled internal consistency of the scale, and the results indicated good reliability (Cronbach’s *α* = 0.872). Furthermore, given the nested structure of the diary data, with daily observations nested within individuals, multilevel confirmatory factor analysis (MCFA) was conducted to examine the one-factor structure of the scale at both the within-person and between-person levels. The results supported a unidimensional structure at both levels, with good model fit: χ^2^ = 16.913, df = 10, *p* = 0.076, CFI = 0.989, TLI = 0.978, RMSEA = 0.014, SRMRwithin = 0.008, and SRMRbetween = 0.009. Multilevel reliability analysis further showed that the scale demonstrated acceptable to good internal consistency at both the within-person and between-person levels (ω_w_ = 0.800, ω_B_ = 0.961) (Geldhof et al., 2014).

##### 3.3.2.2 Daily design learning self-efficacy

This study used five items to measure participants’ daily beliefs in their ability to complete design learning tasks. The items were developed based on the core concept of self-efficacy theory, namely individuals’ beliefs in their capability to organize and execute specific actions required to attain desired goals (Bandura, 1977, 1997), and with reference to measures of academic self-efficacy concerning learning task completion, problem solving, and perceived control over learning (Usher and Pajares, 2008). The items were adapted to the daily learning context of design students. The scale covered confidence in completing design learning tasks, generating valuable design ideas, solving problems during the design process, improving work based on feedback, and advancing design courses or projects. This operationalization emphasizes design-specific efficacy beliefs rather than general academic confidence. A sample item was: “Today, I believe I can complete my design learning tasks.” All items were rated on a 7-point Likert scale, ranging from 1 (“strongly disagree”) to 7 (“strongly agree”). Daily design learning self-efficacy was calculated as the mean of the five items, with higher scores indicating stronger daily beliefs in one’s ability to complete design learning tasks.

This study first calculated the pooled internal consistency of the scale, which indicated good reliability (Cronbach’s α = 0.876). Because the diary observations were nested within individuals, psychometric properties were further evaluated separately at the within-person and between-person levels. Multilevel confirmatory factor analysis (MCFA) was conducted to examine the factorial validity of the five-item measure across the two levels. The results supported a unidimensional structure, with χ^2^ = 204.066, df = 10, *p* < 0.001, CFI = 0.967, TLI = 0.933, RMSEA = 0.073, SRMRwithin = 0.035, and SRMRbetween = 0.005. The low SRMR values at both levels and the overall pattern of fit indices supported the adequacy of the one-factor structure at both the within-person and between-person levels. Multilevel reliability analysis further showed good internal consistency, with ω_w_ = 0.808 at the within-person level and ω_B_ = 0.955 at the between-person level. In addition, daily design-learning self-efficacy was negatively associated with hassles, consistent with the theoretical expectation that higher task demands are associated with lower learning confidence.

### 3.4 Data analysis

This study used IBM SPSS Statistics 26 for Windows for data cleaning, screening, and descriptive statistical analyses, and Mplus 8.3 for dynamic structural equation modeling of the diary data (Muthén and Muthén, 2017). To examine potential self-selection into the intensive longitudinal phase, we additionally compared the baseline characteristics of students who agreed to participate in the diary phase (*n* = 312) with those who did not participate (n = 788). Gender and grade-level distributions were compared using Pearson Chi-square tests. The main analyses were conducted using a bivariate dynamic structural equation model (Asparouhov et al., 2018; McNeish and Hamaker, 2020). DSEM combines multilevel modeling, time-series modeling, and structural equation modeling within a Bayesian estimation framework, making it suitable for estimating within-person dynamic effects and between-person differences in intensive longitudinal data (Muthén and Asparouhov, 2012).

The bivariate DSEM was estimated using Bayesian Markov chain Monte Carlo (MCMC) estimation. The two repeatedly measured variables were decomposed into within-person and between-person components within the DSEM framework. Accordingly, the within-person component represented fluctuations around each participant’s own typical level across the instructional-day assessments, whereas the between-person component represented stable individual differences in average levels. No additional manual person-mean centering was applied to the repeatedly measured variables. Gender and baseline hassles were entered as time-invariant between-person covariates.

The final model was estimated using 50,000 MCMC iterations and two chains to estimate model parameters and handle missing data (Gelman et al., 2013). No user-specified informative priors were imposed, and the default Bayesian priors implemented in Mplus were used. Missing instructional-day observations were retained in the longitudinal data structure and handled within the Bayesian estimation procedure. Convergence was evaluated using the potential scale reduction factor (PSR), with values approaching 1.00 indicating convergence and values below 1.10 considered acceptable.

Parameter uncertainty was evaluated using 95% Bayesian credible intervals (CrIs), corresponding to the 2.5th and 97.5th percentiles of the posterior distribution. A parameter was considered statistically credible when its 95% CrI did not include zero (McElreath, 2018). Model adequacy was further evaluated using posterior predictive checking. The posterior predictive *p* value (PPP), together with the 95% interval for the difference between the observed and replicated discrepancy statistics, was used to assess the correspondence between the observed data and data replicated from the posterior distribution.

Time was indexed according to successive instructional-day diary assessments. Accordingly, a lag of one represented the interval between one scheduled instructional-day assessment and the subsequent scheduled instructional-day assessment.

The full model specification, including the within-person and between-person components, is presented in Figure 2. In the model, each core variable served simultaneously as both a predictor and an outcome. At the between-person level, this study estimated the overall means of the two variables, namely the fixed effects, as well as between-person variances, namely the random effects. At the within-person level, two autoregressive parameters were first estimated by regressing generative AI use at assessment t (A_it_) and design-learning self-efficacy at assessment t (E_it_) on their respective levels at the preceding instructional-day assessment (A_i,t − 1_ and E_i,t − 1_). In addition, current-day design learning self-efficacy (E_it_) was regressed on generative AI use at the preceding instructional-day assessment in design learning (A_i,t − 1_), representing the cross-lagged association from generative AI use to subsequent design-learning self-efficacy. Generative AI use at assessment t (A_it_) was also regressed on design-learning self-efficacy at the preceding instructional-day assessment (E_i,t − 1_), representing the cross-lagged association from design-learning self-efficacy to subsequent generative AI use. These two paths constituted the cross-lagged effects.

**Figure 2:**
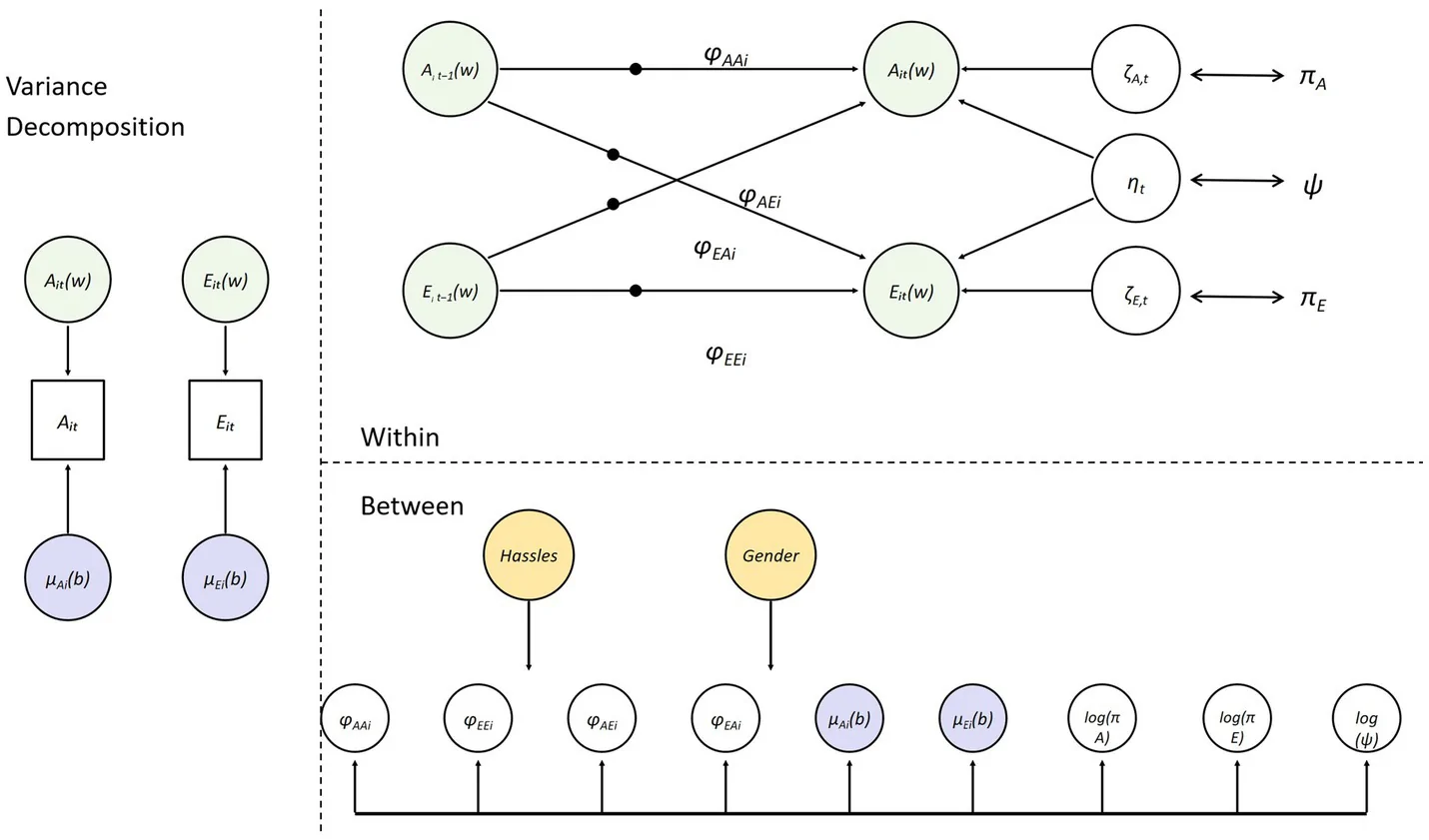
Bivariate dynamic structural equation model of daily generative AI use in design learning (A) and daily design-learning self-efficacy (E). The model separates within-person autoregressive and cross-lagged effects from between-person differences. Gender and baseline hassles were included as between-person covariates.

The above within-person parameters were allowed to vary across individuals, that is, they were treated as random effects, and these random effects were allowed to correlate with one another at the between-person level. Gender was included as a demographic control, and hassles were included as an indicator of students’ task-demand context to predict the random effects. Baseline hassles were treated as a time-invariant between-person covariate and were used to predict individual differences in the random effects. In particular, their association with the random cross-lagged slope from generative AI use to subsequent design-learning self-efficacy was used to examine whether the strength of this within-person dynamic association differed according to students’ baseline level of hassles.

## 4 Results

### 4.1 Descriptive statistics

Before examining the intensive longitudinal results, we compared the available baseline characteristics of students who agreed to participate in the diary phase (*n* = 312) with those who did not participate (n = 788). As shown in Supplementary Table S2, gender composition did not differ significantly between the two groups, χ^2^(1) = 1.527, *p* = 0.217, Cramér’s V = 0.037. Grade-level composition differed significantly, χ^2^(3) = 15.237, *p* = 0.002, although the effect size was small (Cramér’s V = 0.118). Thus, the available baseline comparisons indicated no meaningful difference in gender and small differences in grade composition. Table 2 presents the descriptive statistics, intraclass correlation coefficients, and between- and within-person associations for the two daily study variables. Both variables showed substantial variation at the within-person and between-person levels. Specifically, the ICCs were 0.471 for daily generative AI use and 0.466 for design-learning self-efficacy, indicating that slightly more than half of the variance in both variables reflected within-person daily fluctuations. The two variables were positively associated at both levels, although the between-person association (*r*_B_ = 0.32, *p* < 0.001) was stronger than the concurrent within-person association (*r*_w_ = 0.04, *p* < 0.05).

**Table 2:** Descriptive statistics, intraclass correlations, and between- and within-person associations among the daily study variables.

Variable	M	SD	ICC	1	2
1. Daily generative AI use	4.05	1.12	0.471		0.04 ^*^
2. Design learning self-efficacy	4.21	1.16	0.466	0.32^***^	

*N* = 260 participants. Within-person descriptive statistics and correlations were based on 3,558 observed person-day assessments. ICC, intraclass correlation coefficient; r_B_, between-person correlation; r_w_, within-person correlation. *p < 0.05. ***p < 0.001.

### 4.2 Within-person effects

The Bayesian DSEM showed satisfactory convergence and posterior predictive fit. The potential scale reduction factor (PSR) at the final iteration was 1.035, which was below the criterion of 1.10, indicating acceptable convergence of the MCMC chains. Posterior predictive checking yielded a posterior predictive *p* value (PPP) of 0.533. The 95% interval for the difference between the observed and replicated discrepancy statistics ranged from −333.902 to 298.997 and included zero. Taken together, these diagnostics indicated acceptable posterior predictive model fit. The substantive results are therefore reported below as standardized posterior estimates with 95% Bayesian credible intervals (CrIs).

The primary analysis examined whether deviations from students’ usual levels of generative AI use and design-learning self-efficacy predicted subsequent deviations in the other variable across successive instructional days. As shown in Table 3, generative AI use at the previous instructional-day assessment significantly and positively predicted design-learning self-efficacy at the subsequent instructional-day assessment (*β* = 0.043, 95% CrI [0.006, 0.077]). This indicates that when an individual’s generative AI use was one within-person standard deviation higher than their own average level on a given instructional day, their design-learning self-efficacy at the subsequent instructional-day assessment increased by 0.043 within-person standard deviations on average. Similarly, design-learning self-efficacy at the previous instructional-day assessment significantly and positively predicted generative AI use at the subsequent instructional-day assessment (*β* = 0.059, 95% CrI [0.023, 0.095]). Because the cross-lagged effect of design learning self-efficacy on subsequent generative AI use was slightly larger than the effect of generative AI use on subsequent design learning self-efficacy (0.059 vs. 0.043), the results indicate a small but statistically credible reciprocal association between the two variables, with design learning self-efficacy showing a slightly stronger association with subsequent generative AI use.

**Table 3:** Standardized within-person effects for the bivariate DSEM.

Predictor	Outcome	Notation	Estimate	SE	*p*	95% CrI Lower	95% CrI Upper
A_t-1_	A_t_	φ_AA,i_	0.198	0.021	0.000	0.154	0.237
E_t-1_	E_t_	φ_EE,i_	0.138	0.019	0.000	0.100	0.175
A_t-1_	E_t_	φ_AE,i_	0.043	0.018	0.010	0.006	0.077
E_t-1_	A_t_	φ_EA,i_	0.059	0.019	0.001	0.023	0.095

A, daily generative AI design learning use; E, daily design learning self-efficacy; i, individual; t, time point, that is, the specific day the measurement occurred; φ_AA,i_, autoregressive effect of generative AI design learning use; φ_EE,i_, autoregressive effect of design learning self-efficacy; φ_AE,i_, cross-lagged effect of generative AI design learning use on design learning self-efficacy; φ_EA,i_, cross-lagged effect of design learning self-efficacy on generative AI design learning use.

Autoregressive effects reflect the continuity of variables across adjacent time points and can also be understood as inertia or carryover effects. As shown in Table 3, daily generative AI use in design learning showed a statistically credible autoregressive effect (*β* = 0.198, 95% CrI [0.154, 0.237]), indicating that higher generative AI use at the preceding instructional-day assessment was associated with higher use at the subsequent instructional-day assessment. Daily design-learning self-efficacy showed a similar autoregressive effect (*β* = 0.138, 95% CrI [0.100, 0.175]), indicating carryover in self-efficacy across successive instructional-day assessments. In comparison, the autoregressive effect of daily generative AI use in design learning was stronger than that of design learning self-efficacy (0.198 vs. 0.138), suggesting that generative AI use exhibited greater continuity in daily learning contexts. Although both cross-lagged paths were statistically credible, their standardized magnitudes were small. Therefore, the results should be interpreted as evidence of a modest subsequent instructional day reciprocal association rather than a strong feedback loop.

### 4.3 Between-person effects (random effects regressed on covariates)

Table 4 reports the results of regressing the random effects on the covariates. First, gender did not significantly predict the between-person mean levels, autoregressive effects, or cross-lagged effects of the two variables. This indicates that, in the present sample, male and female students did not differ significantly in daily generative AI use in design learning, daily design learning self-efficacy, or the dynamic relationship between them.

**Table 4:** Random effects regressed on covariates.

Predictor	Outcome	Estimate	SE	*p*	95% CrI Lower	95% CrI Upper
Gender	μ_A,i_	−0.015	0.046	0.370	−0.105	0.080
	μ_E,i_	−0.019	0.043	0.352	−0.108	0.064
	φ_AA,i_	−0.032	0.172	0.426	−0.394	0.306
	φ_EE,i_	−0.032	0.161	0.411	−0.380	0.251
	φ_AE,i_	0.255	0.169	0.053	−0.033	0.635
	φ_EA,i_	0.163	0.169	0.133	−0.151	0.522
Hassles	μ_A,i_	0.135	0.045	0.003	0.049	0.222
	μ_E,i_	−0.254	0.043	0.000	−0.332	−0.164
	φ_AA,i_	−0.400	0.203	0.005	−0.840	−0.093
	φ_EE,i_	0.074	0.177	0.297	−0.233	0.548
	φ_AE,i_	0.458	0.167	0.002	0.149	0.785
	φ_EA,i_	0.103	0.151	0.246	−0.166	0.434

For gender, female was coded 1 and male was coded 2; each predictor has these variables as outcomes, that is, μ_A,i_, μ_E,i_, φ_AA,i_, φ_EE,i_, φ_AE,i_, and φ_EA,i_. A, daily generative AI design learning use; E, daily design learning self-efficacy; i, individual; t, time point; μ, mean; μ_A,i_, between-person mean level of daily generative AI design learning use; μ_E,i_, between-person mean level of daily design learning self-efficacy; φ_AA,i_, autoregressive effect of daily generative AI design learning use; φ_EE,i_, autoregressive effect of daily design learning self-efficacy; φ_AE,i_, cross-lagged effect of generative AI design learning use on design learning self-efficacy; φ_EA,i_, cross-lagged effect of design learning self-efficacy on generative AI design learning use.

Second, hassles significantly predicted several random effects. Specifically, hassles significantly and positively predicted the between-person mean level of daily generative AI use in design learning (*β* = 0.135, 95% CrI [0.049, 0.222]), indicating that students with higher levels of hassles were generally more likely to use generative AI in their daily design learning. At the same time, hassles significantly and negatively predicted the between-person mean level of daily design learning self-efficacy (*β* = −0.254, 95% CrI [−0.332, −0.164]), suggesting that students who experienced more learning-related hassles generally reported lower design learning self-efficacy.

Regarding the dynamic effects, hassles significantly and negatively predicted the autoregressive effect of generative AI use in design learning (*β* = −0.400, 95% CrI [−0.840, −0.093]). This indicates that, for students experiencing higher levels of learning-related hassles, the continuity across successive instructional days of generative AI use was weaker, suggesting that their use behavior may be more susceptible to daily task contexts. Hassles also significantly and positively predicted the cross-lagged effect from generative AI use to subsequent design-learning self-efficacy (*β* = 0.458, 95% CrI [0.149, 0.785]), indicating that, among students with higher baseline hassles, generative AI use at the preceding instructional-day assessment was more strongly associated with design-learning self-efficacy at the subsequent instructional-day assessment. In contrast, hassles did not significantly predict the autoregressive effect of design-learning self-efficacy or the cross-lagged effect from design-learning self-efficacy to subsequent generative AI use.

## 5 Discussion

This study aimed to examine the daily dynamic relationship between daily generative AI use in design learning and daily design learning self-efficacy among Chinese university students majoring in design. Based on 14-instructional-day diary data, this study used a bivariate dynamic structural equation model (DSEM) to distinguish within-person daily fluctuation processes from stable between-person differences. The results showed that, after controlling for between-person differences in mean levels and relevant covariates, daily generative AI use in design learning and daily design learning self-efficacy had a significant bidirectional predictive relationship at the within-person level. Specifically, students’ higher level of generative AI use on a given instructional day predicted higher design learning self-efficacy on the subsequent instructional day, while their higher level of design learning self-efficacy on a given instructional day also predicted higher generative AI use on the subsequent instructional day. These findings extend previous research on generative AI use, learning beliefs, and technology adoption by showing that their association is not only a between-person phenomenon but also appears in within-person fluctuations across consecutive learning days. Rather than indicating a strong feedback loop, the results point to a modest reciprocal pattern in which generative AI use and design learning self-efficacy may support each other over time.

### 5.1 Within-person effects

On the one hand, this study found that daily generative AI use in design learning significantly predicted on the subsequent instructional day design learning self-efficacy. This finding suggests that generative AI use is not merely an external technical aid for completing design tasks, but may also be transformed in the short term into students’ positive judgments of their own design learning capabilities. Previous research has shown that university students generally view generative AI as a learning tool that can support personalized learning, brainstorming, writing, information integration, and research analysis (Chan and Hu, 2023). In the context of design learning, this role may be even more pronounced. Design learning tasks are typically open-ended, ambiguous, and iterative, requiring students to move continuously through ideation, alternative comparison, visual expression, feedback-based revision, and work refinement. Generative AI can provide immediate feedback, diverse references, and low-threshold support for solution generation, thereby reducing uncertainty in the early stages of design and offering students more actionable pathways for task progress. Existing research on the educational applications of large language models has also noted the potential of generative AI to provide personalized support, facilitate learning feedback, and assist in the understanding of complex tasks (Crompton and Burke, 2023; Kasneci et al., 2023). Therefore, when students use generative AI more frequently to support design learning on a given instructional day, they may be more likely to develop a sense of capability and control in advancing design tasks on the subsequent instructional day.

The positive association between generative AI use and subsequent design-learning self-efficacy should not be interpreted as indicating that all forms of AI use are equally beneficial. The present measure captured task-related engagement but did not distinguish reflective, evaluative, and transformative use from superficial reliance or uncritical acceptance of AI-generated outputs. In design learning, these forms of engagement may have substantially different educational consequences. Productive AI-supported learning is more likely to involve comparing alternatives, evaluating generated content, revising outputs, and integrating AI suggestions into students’ own design reasoning, whereas superficial reliance may increase perceived task ease or confidence without necessarily strengthening design competence. Therefore, the present findings concern the extent of task-related AI engagement rather than the pedagogical quality of that engagement.

This finding can also be explained by self-efficacy theory. According to Bandura, self-efficacy is mainly derived from sources such as mastery experiences, vicarious experiences, social persuasion, and emotional states (Bandura, 1977, 1997). The role of generative AI in design learning may correspond closely to these sources of efficacy. It can help students produce initial solutions more quickly, thereby creating partial mastery experiences; provide multiple design examples and visual references, thereby offering vicarious experiences; strengthen students’ perceived task controllability through immediate feedback and revision suggestions; and alleviate anxiety and frustration in design learning by reducing ideation blocks and task uncertainty. Thus, this study extends previous discussions of the learning-support role of generative AI to the within-person daily dynamic level, showing that after students use generative AI at a higher level on a given instructional day, their design learning self-efficacy shows a small but significant increase on the subsequent instructional day.

On the other hand, this study also found that daily design learning self-efficacy significantly predicted on the subsequent instructional day generative AI use in design learning. This finding indicates that generative AI use is not necessarily only a passive compensatory behavior when students feel less capable, experience higher pressure, or encounter task obstacles. It may also be an active learning strategy driven by positive learning beliefs. In other words, when students believe more strongly than usual on a given instructional day that they can complete design learning tasks, they may be more willing the next day to actively explore generative AI tools, try different prompts, compare AI-generated results, and integrate AI outputs into their own design thinking processes. Existing research on technology acceptance and generative AI use has shown that students’ positive judgments of technological value, learning benefits, and their own capabilities influence their use intentions and learning engagement (Chan and Zhou, 2023; Getenet et al., 2024). The present study further demonstrates that this relationship exists not only at the level of between-person differences but also at the level of daily fluctuations within the same student: subsequent instructional days on which students’ self-efficacy is higher than their own average level, their generative AI use is also more likely to be above their usual level.

Notably, this study found that the cross-lagged effects in both directions were significant but small in magnitude. The predictive effect of design learning self-efficacy on the subsequent instructional day generative AI use (β = 0.059) was slightly larger than that of generative AI use on the subsequent instructional day design learning self-efficacy (β = 0.043). This result suggests that, in daily design learning, students’ positive beliefs about their own capabilities may serve as an important psychological foundation for their continued exploration of AI tools. At the same time, the immediate support provided by AI use may in turn enhance their subsequent design learning self-efficacy. Taken together, these findings are better interpreted as a modest reciprocal process than as a strong self-reinforcing cycle.

The small magnitude of the two cross-lagged effects is also theoretically informative. Daily design-learning self-efficacy is unlikely to be determined by a single source of support, because students’ confidence may simultaneously depend on task difficulty, feedback quality, recent success or failure, time pressure, and emotional experience. Similarly, daily generative AI use may be shaped by immediate task requirements, assignment stages, tool familiarity, and students’ willingness to experiment with AI. Therefore, the relatively small subsequent instructional day effects observed here are consistent with the view that generative AI use and self-efficacy represent only one part of a broader daily learning system. The slightly larger effect from self-efficacy to subsequent AI use (*β* = 0.059) than from AI use to subsequent self-efficacy (*β* = 0.043) further suggests that efficacy beliefs may function not only as an outcome of AI-supported learning but also as a psychological condition that facilitates subsequent engagement with AI tools. However, given the small difference between the two effects, this asymmetry should be interpreted cautiously rather than as evidence that one direction is clearly dominant. Another important finding concerns the difference between the between-person and within-person associations. The between-person correlation between generative AI use and design-learning self-efficacy was moderate (*r_B_* = 0.32), whereas the concurrent within-person correlation was much smaller (*r_w_* = 0.04). This difference indicates that students who generally use generative AI more frequently also tend to report higher overall self-efficacy, but this between-person pattern should not be interpreted as meaning that increases in AI use on a particular day are accompanied by equally large increases in self-efficacy within the same student. This distinction is consistent with methodological arguments that between-person associations and within-person processes represent different levels of explanation and should not be treated as interchangeable (Curran and Bauer, 2011; Molenaar, 2004). The significant but small cross-lagged effects found in the present study therefore add information beyond the between-person correlation by showing that, after separating stable individual differences, deviations from a student’s usual level of one variable are modestly associated with the other variable on the subsequent instructional day.

In addition, this study found that both daily generative AI use in design learning and daily design learning self-efficacy showed significant autoregressive effects. Autoregressive effects generally reflect the continuity of variables across adjacent time points and can also be understood as inertia or carryover in individual states (Ployhart et al., 2025). Specifically, the autoregressive effect of daily generative AI use in design learning was β = 0.198, indicating that students’ AI use on the subsequent instructional day significantly predicted their use on the subsequent instructional day. The autoregressive effect of daily design learning self-efficacy was *β* = 0.138, indicating that students’ design learning self-efficacy on the subsequent instructional day also carried over to the subsequent instructional day. In comparison, generative AI use showed stronger inertia than design learning self-efficacy, suggesting that once AI use becomes embedded in students’ daily design learning routines, it may be more likely to develop into a continuous learning habit or tool-dependence pathway. By contrast, although design learning self-efficacy also showed some stability, it may be more susceptible to contextual factors such as daily course tasks, feedback on design work, design progress, and emotional experiences.

### 5.2 Between-person differences and the contextual role of baseline hassles

Based on prior research on technology acceptance and digital learning, gender has often been regarded as an important demographic variable influencing technology attitudes, technological self-efficacy, and technology use intentions (Scherer et al., 2019; Venkatesh et al., 2012). However, unlike some previous studies, the present study did not find that gender significantly predicted the between-person mean levels of daily generative AI use in design learning or daily design learning self-efficacy. At the same time, gender did not significantly predict the autoregressive or cross-lagged effects of the two variables. These results indicate that, in the present sample, male and female students did not differ substantially in their overall levels of generative AI use or design learning self-efficacy, nor did they show significant differences in the daily dynamic relationship between the two variables. This may be related to the low threshold for using generative AI tools and the common task demands in design learning. Compared with traditional professional software, generative AI typically relies on natural language prompts, image generation, and immediate feedback as its main modes of interaction. Therefore, differences in its use may no longer be primarily explained by gender, but may instead be more strongly influenced by task context, AI literacy, tool familiarity, and learning strategies (Chan and Hu, 2023; Crompton and Burke, 2023; Getenet et al., 2024).

In contrast to the findings for gender, this study found that hassles explained some between-person differences. First, hassles significantly and positively predicted the between-person mean level of daily generative AI use in design learning. This indicates that students experiencing higher higher baseline hassles were generally more likely to use generative AI to support their design learning. This finding is consistent with the learning-support function of generative AI in higher education. Previous studies have shown that students use generative AI for brainstorming, information integration, feedback acquisition, text and visual content generation, and the handling of complex learning tasks (Chan and Hu, 2023; Crompton and Burke, 2023; Kasneci et al., 2023). For design students, high task demands often involve greater ideation pressure, solution iteration, visual expression, work revision, and deadline pressure. In such situations, generative AI may be viewed by students as a tool for reducing cognitive load, expanding design thinking, and improving task progress efficiency (Jia and Tu, 2024; Sweller, 1988).

Second, this study found that hassles significantly and negatively predicted the between-person mean level of daily design learning self-efficacy. This suggests that students experiencing higher higher baseline hassles were generally less confident in their ability to complete design learning tasks effectively. This finding is consistent with self-efficacy theory. According to Bandura, self-efficacy is shaped by information sources such as mastery experiences, emotional states, task obstacles, and social feedback (Bandura, 1977, 1997). When students are chronically exposed to higher design task pressure, ambiguous assessment criteria, and repeated unsuccessful revisions, their judgments of their ability to complete design tasks may decline (Hartelt and Martens, 2024). Therefore, it is understandable that students with higher levels of hassles showed lower design learning self-efficacy.

In addition, this study found that hassles significantly and negatively predicted the autoregressive effect of generative AI use in design learning. This indicates that although students with higher levels of learning-related hassles were generally more likely to use generative AI, the continuity across successive instructional days of their AI use was weaker. In other words, their AI use may be more context-driven rather than habitually stable. When design task pressure is high or when they encounter specific design difficulties on a given instructional day, these students may rely on AI more frequently; however, when task pressure decreases or the task stage changes, their level of AI use may also decline. This interpretation is consistent with cognitive load theory, which suggests that learners are more likely to seek external tool support when task demands are high, in order to reduce internal processing pressure and maintain task progress (Sweller, 1988).

Finally, hassles significantly and positively predicted the cross-lagged effect from generative AI use to subsequent design-learning self-efficacy. This result indicates that, among students with higher baseline hassles, generative AI use at the preceding instructional-day assessment was more strongly associated with design-learning self-efficacy at the subsequent instructional-day assessment. This finding further reveals a boundary condition of the learning-support role of generative AI: generative AI may not function as a general tool that exerts the same influence on all students, but may provide more pronounced psychological support in high-task-demand contexts. For students facing greater design learning pressure, the functions of generative AI, such as inspiration generation, solution comparison, visual referencing, and feedback-based optimization, may be more readily transformed into a sense of task control and efficacy beliefs. From a dynamic perspective, this result extends previous research on generative AI-supported learning by showing that hassles not only influence students’ average levels of AI use and self-efficacy, but also alter the daily dynamic link between the two variables.

Taken together, the pattern associated with baseline hassles is more informative when the significant effects are considered jointly rather than separately. Students with higher baseline hassles reported higher average generative AI use but lower average design-learning self-efficacy. At the same time, their AI use showed weaker stability across successive instructional days, while the cross-lagged association from AI use to subsequent self-efficacy was stronger. This combination suggests a context-sensitive rather than purely habitual pattern of AI use. Students experiencing greater background task demands may rely on generative AI more frequently overall, but their use may also fluctuate more strongly according to immediate task needs. When such students engage with generative AI, the resulting task support may be more strongly associated with subsequent efficacy beliefs because external assistance is especially salient when students have fewer psychological or task resources available. This interpretation is consistent with the idea that generative AI may function as a compensatory learning resource under demanding conditions rather than exerting an equally strong effect for all students. Importantly, because hassles were assessed once at baseline rather than on each diary day, these findings should be interpreted as between-person differences in the broader task-demand context; they do not demonstrate that day-specific increases in hassles directly caused students to use AI more or become more responsive to AI support.

## 6 Conclusions and implications

### 6.1 Conclusion

This study used a 14-instructional-day diary design and bivariate DSEM to examine the reciprocal associations between generative AI use in design learning and design-learning self-efficacy across successive instructional days among Chinese undergraduate design students. The findings showed a modest but statistically credible reciprocal pattern: higher-than-usual generative AI use on one instructional day predicted slightly higher design-learning self-efficacy on the subsequent instructional day (*β* = 0.043), while higher-than-usual design-learning self-efficacy on one instructional day predicted slightly greater generative AI use on the subsequent instructional day (*β* = 0.059). Baseline hassles were associated with higher average generative AI use, lower average design-learning self-efficacy, weaker stability in generative AI use across successive instructional days, and a stronger cross-lagged association from generative AI use to subsequent design-learning self-efficacy. Gender did not significantly predict the between-person mean levels, autoregressive effects, or cross-lagged effects of the two focal variables. Overall, these findings suggest that generative AI use and design-learning self-efficacy are dynamically linked across successive instructional days, although the magnitude of the reciprocal associations is small. These findings should therefore be interpreted as temporal predictive associations rather than evidence of causal effects or improvements in objective design performance.

### 6.2 Theoretical implications

This study offers three theoretical contributions. First, it advances research on generative AI in education from static associations to daily dynamic processes. Existing studies have mostly examined students’ attitudes toward generative AI, usage intentions, perceived learning benefits, or learning performance from a cross-sectional perspective, with limited attention to how generative AI use and learning-related psychological states influence each other within the same student across consecutive daily learning contexts. Based on 14-instructional-day diary data, this study found a significant bidirectional cross-lagged relationship between generative AI use in design learning and design learning self-efficacy. This suggests that the relationship between the two is not merely a between-person difference, but may also appear as a modest within-person reciprocal pattern across consecutive learning days.

Second, this study extends self-efficacy theory to the context of generative AI-supported learning. Self-efficacy theory emphasizes that individuals’ beliefs about their capabilities are shaped by information sources such as mastery experiences, vicarious experiences, social feedback, and emotional states. The findings indicate that, in design learning contexts, generative AI may become an important contextual resource influencing students’ daily self-efficacy by providing inspiration, feedback, solution comparison, and task-progress support. At the same time, design learning self-efficacy also predicts students’ subsequent generative AI use. This result suggests that students’ capability beliefs and technology use behaviors may be reciprocally linked over short time intervals, rather than operating only in a single direction.

Third, by using DSEM to distinguish between-person differences from within-person changes, this study provides a useful methodological paradigm for research on generative AI in education. Traditional cross-sectional studies can typically answer only whether students who use generative AI more frequently have higher self-efficacy, but cannot determine whether the same student’s self-efficacy increases on the subsequent instructional day after using AI more than usual. By applying a bivariate DSEM, this study simultaneously estimated autoregressive effects, cross-lagged effects, and the explanatory role of between-person covariates in random effects, thereby revealing the dynamic mechanism between generative AI use and learning-related psychological states more precisely. This provides a methodological reference for future studies examining daily cyclical relationships among generative AI use, learning motivation, learning anxiety, creative confidence, and learning engagement.

### 6.3 Practical implications

Although this study has certain limitations, its findings offer several implications for design education practice. First, this study used a 14-instructional-day diary design to examine the within-person dynamic process between generative AI use in design learning and design learning self-efficacy. Compared with a one-time cross-sectional survey, diary research can more closely capture students’ daily experiences in real learning contexts and, to some extent, reduce recall bias. Therefore, universities and design educators may draw on this dynamic assessment approach by focusing not only on whether students use generative AI, but also on how they use AI across different learning stages, task pressures, and design progress, as well as how such use influences their learning confidence and sense of task control.

Second, the findings show that generative AI use positively predicts subsequent design learning self-efficacy. This means that universities and design educators should not regard generative AI merely as an external technical tool or a potential academic risk, but should also recognize its potential value in supporting design ideation, reducing task uncertainty, and enhancing learning confidence. Teachers may conditionally integrate generative AI into design courses, for example, by guiding students to use AI for early-stage brainstorming, visual reference collection, solution comparison, textual explanation refinement, and iterative feedback on design work. At the same time, teachers should require students to explain how AI outputs are evaluated, selected, revised, and transformed into their own design decisions, so as to avoid passive dependence on AI-generated results. Educators should also distinguish between AI-supported confidence and genuine design competence, because increased self-efficacy does not necessarily indicate improved design performance.

Third, this study found that design learning self-efficacy also positively predicts subsequent generative AI use. Thus, in design education, enhancing students’ capability beliefs may help promote more active and proactive use of generative AI. Teachers can help students accumulate mastery experiences through staged feedback, formative assessment, peer presentation, and small-step task design. When students believe that they can complete design tasks, they may be more willing to explore AI tools, refine prompts, compare different generated solutions, and use AI as a collaborative resource for design thinking rather than as a simple substitute tool.

Finally, this study found that students with higher levels of hassles were more likely to use generative AI, and that among these students, generative AI use showed a stronger positive association with subsequent design learning self-efficacy. This suggests that teachers should pay particular attention to how students with high task loads use AI. For these students, teachers can provide clearer AI use guidelines, process documentation requirements, and evaluation criteria, such as requiring students to retain prompts, generated outputs, revision processes, and explanations of final design decisions. This can help students use AI to reduce task pressure and improve learning confidence, while also reducing inappropriate use, overreliance, and academic integrity risks. Overall, the value of generative AI in design education should not be understood only from the perspective of tool efficiency, but should also be designed and managed from the perspectives of learning support, psychological empowerment, and process-oriented guidance.

## 7 Limitations and future research

First, this study mainly relied on students’ self-reported data, which may be subject to common method bias, recall bias, and social desirability bias. In particular, daily generative AI use was assessed as self-reported task-oriented engagement rather than through objective indicators such as usage duration, number of interactions, or platform-level usage logs. Moreover, the present study did not collect objective learning indicators such as expert-rated design quality, revision behavior, or instructor assessment. Therefore, the present findings should be interpreted as evidence of dynamic associations between students’ self-reported generative AI use and perceived design-learning self-efficacy, rather than as evidence that generative AI use directly improves objective design performance. Future research could incorporate platform usage logs, prompt records, revision traces of design works, learning management system data, instructor evaluations, or independent expert ratings of design quality to capture students’ AI use behaviors and design learning outcomes more objectively. Future studies should also distinguish between frequency, duration, quality, purpose, and depth of generative AI use, as self-reported use may not fully reflect how students evaluate and transform AI-generated outputs. In particular, future intensive longitudinal measures could separately assess reflective and productive behaviors, such as evaluating, comparing, revising, and transforming AI-generated content, and superficial reliance behaviors, such as direct copying or uncritical acceptance of generated outputs.

Second, although self-efficacy theory provided the principal theoretical framework for this study, the mechanisms proposed by the theory were not directly measured. Specifically, the study did not separately assess mastery experiences, vicarious experiences, social persuasion, or emotional states as mediating processes through which generative AI use might become associated with subsequent self-efficacy. Consequently, the interpretation that generative AI may contribute to efficacy beliefs through task progress, feedback, or reduced uncertainty remains theoretically plausible but was not directly tested. Future research should explicitly measure these efficacy-related mechanisms and examine whether they mediate the subsequent instructional day association between generative AI use and design-learning self-efficacy.

Third, baseline hassles were assessed once before the diary phase and were treated as a time-invariant between-person contextual variable. This design allowed us to examine whether students with different levels of background task demands differed in their average states and dynamic associations, but it cannot determine whether day-specific fluctuations in hassles altered AI use or self-efficacy on particular days. Thus, the observed moderation by baseline hassles should be interpreted as a between-person contextual difference rather than as evidence of a daily stress process. Future diary studies should distinguish between relatively stable background hassles and day-specific stressors by assessing both levels simultaneously.

Fourth, this study adopted a 14-instructional-day diary design, which allowed us to capture short-term dynamic relationships across successive instructional days but could not reveal developmental changes across a full semester or a complete design-project cycle. The lag-1 specification represents associations between successive instructional-day assessments. Although weekends were excluded from the diary schedule, the study did not systematically record other day-specific academic events, such as examination periods or major design-project deadlines, which may have influenced students’ learning opportunities and AI use. Future research could incorporate these contextual factors as time-varying information and employ longer observation periods to examine whether the reciprocal associations vary across different stages of a semester or design project.

Fifth, the bivariate DSEM focused on generative AI use and design-learning self-efficacy, with gender and baseline hassles included as between-person covariates. This parsimonious model helped isolate the reciprocal association between the two focal variables, but it cannot rule out the influence of other time-varying or person-level factors. Variables such as task difficulty, feedback quality, AI literacy, course demands, learning engagement, cognitive load, and creative self-efficacy may contribute to both AI use and self-efficacy. Future research should incorporate these theoretically relevant variables into multivariate intensive longitudinal models to test more comprehensive dynamic mechanisms. Although gender was included as between-person covariates, other potentially relevant individual-difference variables were not incorporated into the present model. In addition, participation in the intensive longitudinal phase was voluntary. The additional baseline comparison showed no meaningful difference in gender and only small differences in grade composition and baseline hassles between diary participants and non-participants; however, self-selection on unmeasured characteristics cannot be ruled out. In particular, baseline generative AI use, design-learning self-efficacy, learning motivation, AI literacy, and willingness to participate in repeated assessments were not available for comparison across the full baseline sample. Therefore, the present findings should be interpreted as characterizing the participating design students rather than as population-representative estimates. Future research should prospectively assess these characteristics across the full recruitment pool to examine potential selection mechanisms more directly.

## Data Availability

The original contributions presented in the study are included in the article/Supplementary material, further inquiries can be directed to the corresponding author.

## References

[ref1] Asparouhov T. Hamaker E. L. Muthén B. (2018). Dynamic structural equation models. Struct. Equ. Model. 25, 359–388. doi: 10.1080/10705511.2017.1406803

[ref2] Bandura A. (1977). Self-efficacy: toward a unifying theory of behavioral change. Psychol. Rev. 84, 191–215. doi: 10.1037//0033-295x.84.2.191 847061

[ref3] Bandura A. (1997). Self-Efficacy: The Exercise of Control. Macmillan.

[ref4] Cena E. McParland A. Toivo W. Dalton B. Mundy M. O’Connor P. A. . (2026). Studying with GenAI: student views on the opportunities and risks of GenAI in higher education. Educ. Inf. Technol. 31, 3655–3681. doi: 10.1007/s10639-026-13923-3

[ref5] Chan C. K. Y. Hu W. (2023). Students’ voices on generative AI: perceptions, benefits, and challenges in higher education. Int. J. Educ. Technol. High. Educ. 20:43. doi: 10.1186/s41239-023-00411-8

[ref6] Chan C. K. Y. Zhou W. (2023). An expectancy value theory (EVT) based instrument for measuring student perceptions of generative AI. Smart Learn. Environ. 10:64. doi: 10.1186/s40561-023-00284-4

[ref7] Chu Y. Li J. Liu S. Liu Y. Xu J. (2025). Differential effects of short- and long-term negative affect on smartphone usage: the moderating role of locus of control. Behav Sci 15:1121. doi: 10.3390/bs15081121, 40867478 PMC12382957

[ref8] Cotton D. R. E. Cotton P. A. Shipway J. R. (2024). Chatting and cheating: ensuring academic integrity in the era of ChatGPT. Innov. Educ. Teach. Int. 61, 228–239. doi: 10.1080/14703297.2023.2190148

[ref9] Crompton H. Burke D. (2023). Artificial intelligence in higher education: the state of the field. Int. J. Educ. Technol. High. Educ. 20:22. doi: 10.1186/s41239-023-00392-8

[ref10] Cross N. (2001). Designerly ways of knowing: design discipline versus design science. Des. Issues 17, 49–55. doi: 10.1162/074793601750357196

[ref11] Curran P. J. Bauer D. J. (2011). The disaggregation of within-person and between-person effects in longitudinal models of change. Annu. Rev. Psychol. 62, 583–619. doi: 10.1146/annurev.psych.093008.100356 PMC3059070 19575624

[ref12] Dorst K. (2011). The core of ‘design thinking’ and its application. Design Studies, Interpreting Design Thinking 32, 521–532. doi: 10.1016/j.destud.2011.07.006

[ref13] Farinosi M. Melchior C. (2025). To adopt or to ban? Student perceptions and use of generative AI in higher education. Humanit. Soc. Sci. Commun. 12:1684. doi: 10.1057/s41599-025-05982-7

[ref14] Farrokhnia M. Banihashem S. K. Noroozi O. Wals A. (2024). A SWOT analysis of ChatGPT: implications for educational practice and research. Innov. Educ. Teach. Int. 61, 460–474. doi: 10.1080/14703297.2023.2195846

[ref15] Geldhof G. J. Preacher K. J. Zyphur M. J. (2014). Reliability estimation in a multilevel confirmatory factor analysis framework. Psychol. Methods 19, 72–91. doi: 10.1037/a0032138, 23646988

[ref16] Gelman A. Carlin J. B. Stern H. S. Rubin D. B. (2013). Bayesian Data Analysis. Chapman and Hall/CRC.

[ref17] Getenet S. Cantle R. Redmond P. Albion P. (2024). Students’ digital technology attitude, literacy and self-efficacy and their effect on online learning engagement. Int. J. Educ. Technol. High. Educ. 21:3. doi: 10.1186/s41239-023-00437-y

[ref19] Hartelt T. Martens H. (2024). Self-regulatory and metacognitive instruction regarding student conceptions: influence on students’ self-efficacy and cognitive load. Front. Psychol. 15:1450947. doi: 10.3389/fpsyg.2024.1450947, 39502150 PMC11534677

[ref20] Honicke T. Broadbent J. (2016). The influence of academic self-efficacy on academic performance: a systematic review. Educ. Res. Rev. 17, 63–84. doi: 10.1016/j.edurev.2015.11.002

[ref21] Huang Y. Hu X. Tian Y. Song Y. Pan Y. (2025). Self-shape congruency in the metaverse: how avatar geometry modulates emotional engagement and virtual consumption. Comput. Hum. Behav. 173:108788. doi: 10.1016/j.chb.2025.108788

[ref22] Hwang Y. Wu Y. (2025). The influence of generative artificial intelligence on creative cognition of design students: a chain mediation model of self-efficacy and anxiety. Front. Psychol. 15:1455015. doi: 10.3389/fpsyg.2024.1455015, 39931512 PMC11808137

[ref23] Jeon J. Lee S. (2023). Large language models in education: a focus on the complementary relationship between human teachers and ChatGPT. Educ. Inf. Technol. 28, 15873–15892. doi: 10.1007/s10639-023-11834-1

[ref24] Jia X.-H. Tu J.-C. (2024). Towards a new conceptual model of AI-enhanced learning for college students: the roles of artificial intelligence capabilities, general self-efficacy, learning motivation, and critical thinking awareness. Systems 12:74. doi: 10.3390/systems12030074

[ref25] Jiang Q. Chen Z. Zhang Z. Zuo C. (2023). Investigating links between internet literacy, internet use, and internet addiction among Chinese youth and adolescents in the digital age. Front. Psych. 14:1233303. doi: 10.3389/fpsyt.2023.1233303, 37743978 PMC10513100

[ref26] Jiang Q. Zhang Y. Wei W. Gu C. (2024). Evaluating technological and instructional factors influencing the acceptance of AIGC-assisted design courses. Comput. Educ.: Artif. Intell. 7:100287. doi: 10.1016/j.caeai.2024.100287

[ref27] Kasneci E. Sessler K. Küchemann S. Bannert M. Dementieva D. Fischer F. . (2023). ChatGPT for good? On opportunities and challenges of large language models for education. Learn. Individ. Differ. 103:102274. doi: 10.1016/j.lindif.2023.102274

[ref28] Kohn P. M. Lafreniere K. Gurevich M. (1990). The inventory of college students’ recent life experiences: a decontaminated hassles scale for a special population. J. Behav. Med. 13, 619–630. doi: 10.1007/BF00844738, 2077142

[ref29] Liu X. Li Y. Cao X. (2024). Bidirectional reduction effects of perceived stress and general self-efficacy among college students: a cross-lagged study. Humanit. Soc. Sci. Commun. 11:271. doi: 10.1057/s41599-024-02785-0

[ref30] Lo C. K. (2023). What is the impact of ChatGPT on education? A rapid review of the literature. Educ. Sci. 13:410. doi: 10.3390/educsci13040410

[ref31] McElreath R. (2018). Statistical Rethinking: A Bayesian course with Examples in R and Stan. Chapman and Hall/CRC.

[ref32] McNeish D. Hamaker E. L. (2020). A primer on two-level dynamic structural equation models for intensive longitudinal data in Mplus. Psychol. Methods 25, 610–635. doi: 10.1037/met0000250, 31855015

[ref33] Møgelvang A. Bjelland C. Grassini S. Ludvigsen K. (2024). Gender differences in the use of generative artificial intelligence chatbots in higher education: characteristics and consequences. Educ. Sci. 14:1363. doi: 10.3390/educsci14121363

[ref34] Molenaar P. C. M. (2004). A manifesto on psychology as idiographic science: bringing the person back into scientific psychology, this time forever. Meas. Interdiscip. Res. Perspect. 2, 201–218. doi: 10.1207/s15366359mea0204_1

[ref35] Muthén B. Asparouhov T. (2012). Bayesian structural equation modeling: a more flexible representation of substantive theory. Psychol. Methods 17, 313–335. doi: 10.1037/a0026802, 22962886

[ref36] Muthén B. Muthén L. (2017). “Mplus,” in Handbook of Item Response Theory, (Chapman and Hall/CRC), 507–518.

[ref37] Ployhart R. E. Bliese P. D. Strizver S. D. (2025). Intensive longitudinal models. Annu. Rev. Organ. Psychol. Organ. Behav. 12, 343–367. doi: 10.1146/annurev-orgpsych-110622-054803

[ref38] Ravšelj D. Keržič D. Tomaževič N. Umek L. Brezovar N. Iahad N. A. . (2025). Higher education students’ perceptions of ChatGPT: a global study of early reactions. PLoS One 20:e0315011. doi: 10.1371/journal.pone.0315011 PMC11798494 39908277

[ref39] Salhab R. Aboushi (2025). Influence of AI literacy and 21st-century skills on the acceptance of generative artificial intelligence among college students. Front. Educ. 10:1640212. doi: 10.3389/feduc.2025.1640212

[ref40] Scherer R. Siddiq F. Tondeur J. (2019). The technology acceptance model (TAM): a meta-analytic structural equation modeling approach to explaining teachers’ adoption of digital technology in education. Comput. Educ. 128, 13–35. doi: 10.1016/j.compedu.2018.09.009

[ref41] Schunk D. H. DiBenedetto M. K. (2020). Motivation and social cognitive theory. Contemp. Educ. Psychol. 60:101832. doi: 10.1016/j.cedpsych.2019.101832

[ref42] Schutte N. S. Li H. (2025). The role of self-efficacy and curiosity in student use of artificial intelligence (AI). Int. J. Educ. Technol. High. Educ. 22:73. doi: 10.1186/s41239-025-00574-6

[ref43] Sweller J. (1988). Cognitive load during problem solving: effects on learning. Cogn. Sci. 12, 257–285. doi: 10.1207/s15516709cog1202_4

[ref44] Tachon G. Shankland R. Marteau-Chasserieau F. Morgan B. Leys C. Kotsou I. (2021). Gratitude moderates the relation between daily hassles and satisfaction with life in university students. Int. J. Environ. Res. Public Health 18:13005. doi: 10.3390/ijerph182413005, 34948616 PMC8702127

[ref45] Tang C. Shi Y. Jiang F. Chen W. (2026). The combined effects of teachers’ caring behavior and academic self-efficacy on the academic achievement of Chinese undergraduates: a fsQCA approach. BMC Psychol. doi: 10.1186/s40359-026-05227-5 PMC13711777 42934799

[ref46] Tlili A. Shehata B. Adarkwah M. A. Bozkurt A. Hickey D. T. Huang R. . (2023). What if the devil is my guardian angel: ChatGPT as a case study of using chatbots in education. Smart Learn. Environ. 10:15. doi: 10.1186/s40561-023-00237-x

[ref47] Usher E. L. Pajares F. (2008). Sources of self-efficacy in school: critical review of the literature and future directions. Rev. Educ. Res. 78, 751–796. doi: 10.3102/0034654308321456

[ref48] Vail J. Chen C. (2026). Using the technology acceptance model to examine generative AI integration in creative design education: a case study of fashion and interior design studios. Des. J., 1–22. doi: 10.1080/14606925.2026.2654047

[ref49] Vasconcelos L. A. Crilly N. (2016). Inspiration and fixation: questions, methods, findings, and challenges. Des. Stud. 42, 1–32. doi: 10.1016/j.destud.2015.11.001

[ref50] Venkatesh V. Thong J. Y. L. Xu X. (2012). Consumer acceptance and use of information technology: extending the unified theory of acceptance and use of Technology1. MIS Quarterly 36, 157–178. doi: 10.2307/41410412

[ref51] Wang H. He G. Wang E. Wang Y. Wang Z. Zheng M. Q. . (2024). Disentangling the relationship between state self-compassion and state coping self-efficacy using dynamic structural equation modeling. Appl. Psychol. Health Well Being 16, 2381–2396. doi: 10.1111/aphw.12593, 39279407

[ref52] Wang R. Siau K. L. Zhang Z. (2026). AI-driven mental health therapy: a comparative study of emotional clarity and stigma reduction across journaling, human-guided therapy, and AI-guided therapy. Decis. Support. Syst. 207:114696. doi: 10.1016/j.dss.2026.114696

[ref53] Wang Y. Zhang Z. Wang C. Li Z. Law R. Zhang Z. (2026). Too concise to decide? The impact of AI-generated review summaries on tourist decision satisfaction. Tour. Manag. 115:105415. doi: 10.1016/j.tourman.2026.105415

[ref54] Zimmerman B. J. (2000). Self-efficacy: an essential motive to learn. Contemp. Educ. Psychol. 25, 82–91. doi: 10.1006/ceps.1999.1016 10620383

