# Supplementary material for: How generative AI becomes embedded in design learning: a 14-instructional-day intensive longitudinal study of reciprocal dynamics between AI use and self-efficacy
Source: Front Psychol. 2026 Oct 14;17:1975841. doi: 10.3389/fpsyg.2026.1975841 (PMC13713661; doi:10.3389/fpsyg.2026.1975841)
Supplement: Supplementary file 1 [file Supplementary_file_1.docx]

Supplementary Material

**Appendix A**

**Table S1. Diary Completion, Submission Timing, and Response-Pattern Quality Checks**

| **Category** | **Indicators** | **result** |
| --- | --- | --- |
| number of questionnaires | Observed assessments | 3,558 |
| Completion time | Mean completion duration | 6.42 min |
|  | Median completion duration | 6.08 min |
|  | IQR | 4.87–7.51 min |
| Distribution of completion time | <3 min | 3.7% |
|  | 3–10 min | 89.4% |
|  | >10 min | 6.9% |
| Submission time distribution | 20:30–20:40 | 74.8% |
|  | 20:41–21:00 | 18.6% |
|  | after 21:00 | 6.6% |
| Repetitive answer mode | Identical adjacent-day response vectors | 2.1% of available adjacent-day pairs |
|  | Same vector for ≥3 successive assessments | 1 participant (0.4%) |

**Table S2. Baseline comparison between diary participants and non-participants.**

| **Variable** | **Category** | **Diary participants (n = 312)** | | **Non-participants (n = 788)** | | **Test statistic** | **p** | **Effect size** |
| --- | --- | --- | --- | --- | --- | --- | --- | --- |
|  |  | **n / M** | **% / SD** | **n / M** | **% / SD** |  |  |  |
| Gender | Male | 155 | 49.7 | 424 | 53.8 | χ²(1) = 1.527 | .217 | Cramér’s V = .037 |
|  | Female | 157 | 50.3 | 364 | 46.2 |  |  |  |
| Grade | Freshman | 73 | 23.4 | 182 | 23.1 | χ²(3) = 15.237 | .002 | Cramér’s V = .118 |
|  | Sophomore | 102 | 32.7 | 252 | 32.0 |  |  |  |
|  | Junior | 56 | 17.9 | 214 | 27.2 |  |  |  |
|  | Senior | 81 | 26.0 | 140 | 17.8 |  |  |  |
